# Diagnostic tests performance indices: an overview

**DOI:** 10.11613/BM.2025.010101

**Published:** 2025-02-15

**Authors:** Farrokh Habibzadeh

**Affiliations:** Independent Research Consultant, Shiraz, Iran

**Keywords:** diagnostic tests, sensitivity, specificity, predictive value of tests, likelihood functions, Bayes theorem

## Abstract

Diagnostic tests are important means in clinical practice. To assess the performance of a diagnostic test, we commonly need to compare its results to those obtained from a gold standard test. The test sensitivity is the probability of having a positive test in a diseased-patient; the specificity, a negative test result in a disease-free person. However, none of these indices are useful for clinicians who are looking for the inverse probabilities, *i.e.,* the probabilities of the presence and absence of the disease in a person with a positive and negative test result, respectively, the so-called positive and negative predictive values. Likelihood ratios are other performance indices, which are not readily comprehensible to clinicians. There is another index proposed that looks more comprehensible to practicing physicians - the number needed to misdiagnose. It is the number of people who need to be tested in order to find one misdiagnosed (a false positive or a false negative result). For tests with continuous results, it is necessary to set a cut-off point, the choice of which affects the test performance. To arrive at a correct estimation of test performance indices, it is important to use a properly designed study and to consider various aspects that could potentially compromise the validity of the study, including the choice of the gold standard and the population study, among other things. Finally, it may be possible to derive the performance indices of a test solely based on the shape of the distribution of its results in a given group of people.

## Graphical abstract

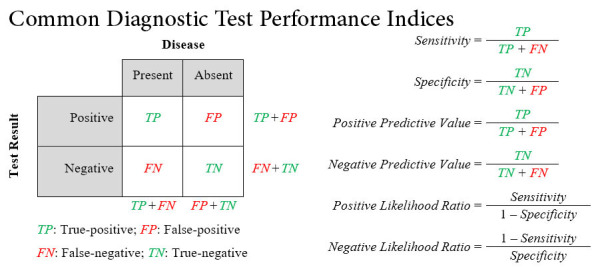

## Highlights

• To assess the performance of a diagnostic test, we commonly compare it against a gold standard test

• The test sensitivity and specificity, positive and negative predictive values, and positive and negative likelihood ratios are among commonly used twin indices used to assess the performance of a test

• The number needed to misdiagnose is a single index that is more comprehensible for clinicians; it is the number of people who need to be tested in order for one to be misdiagnosed by the test

## Introduction

Diagnostic tests are among important tools in clinical medicine and research. For instance, they help physicians with the diagnosis of disease conditions (*1*). When possible, it is better to use gold standard (also termed reference standard, criterion standard, and true standard) tests - tests with no false positive (FP) or false negative (FN) results, by definition (*2*). Nonetheless, it is not always possible; a gold standard test may not exist at all for certain conditions or the test may be hardly accessible or be expensive (*3*). We commonly need to utilize alternative diagnostic tests as surrogates for the gold standards. To better understand the application of various diagnostic tests in clinical medicine, it is important to know how their performance is measured and reported. Herein, I present an overview of the common diagnostic test performance indices.

## Test sensitivity and specificity

While a gold standard test does have neither a FP nor a FN result, most of the diagnostic tests used in practice may result in FP or FN results. To assess the performance of a diagnostic test, we therefore compare its results against those obtained from a gold standard test. Four possible outcomes may occur (Table 1). The test is positive while the disease is really present (the gold standard test is also positive, termed true positive (TP)); the test is negative and there is really no disease (the gold standard test is also negative, termed true negative (TN)); the test is positive while there is really no disease (FP result); and, the test is negative while there is really a disease (FN result). The test sensitivity (Se) is the probability that a diseased-person is test-positive (*1*, *3*, *4*). In mathematical parlance, it is:


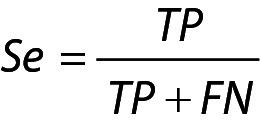
,

**Table 1:** Four possible outcomes when the results of a diagnostic test are compared to the results of a gold standard test

		Disease		
		Present	Absent	
Test Result	Positive	TP	FP	TP + FP
	Negative	FN	TN	FN + TN
		TP + FN	FP + TN	
TP - true positive. FP - false positive. FN - false negative. TN - true negative.				

The Se can be expressed in another way, as a conditional probability:


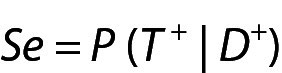
,

The right side of the above equation is a conditional probability and reads “the probability of having a positive test (*T*^+^) given the disease (*D*^+^).” In a similar way, the test specificity (Sp), the probability of a negative test in a person without the disease (*1*, *3*, *4*), is (Table 1):


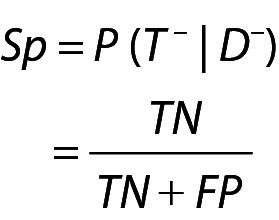
,

A test with a low FN rate has a high Se. Therefore, when a highly sensitive test gives a negative result, it is very unlikely that the result is FN (Eq. 1); it is most probably TN. However, we cannot comment on a positive result of a highly sensitive test; it may be either TP or FP. A highly sensitive test should thus be used to rule out a disease (*e.g.*, for screening purposes); while negative results are important, positive results are not very helpful. Using the same argument, a highly specific test rarely gives a FP result (Eq. 3); a highly specific test should thus be used to rule in a certain disease (*e.g.*, to confirm a diagnosis); while a positive result very likely indicates a disease condition, a negative result is not very helpful (*1*). Note that as a gold standard test results in neither a FP nor FN result, its Se and Sp are 1 (100%).

## Positive and negative predictive values

**Figure 1:**
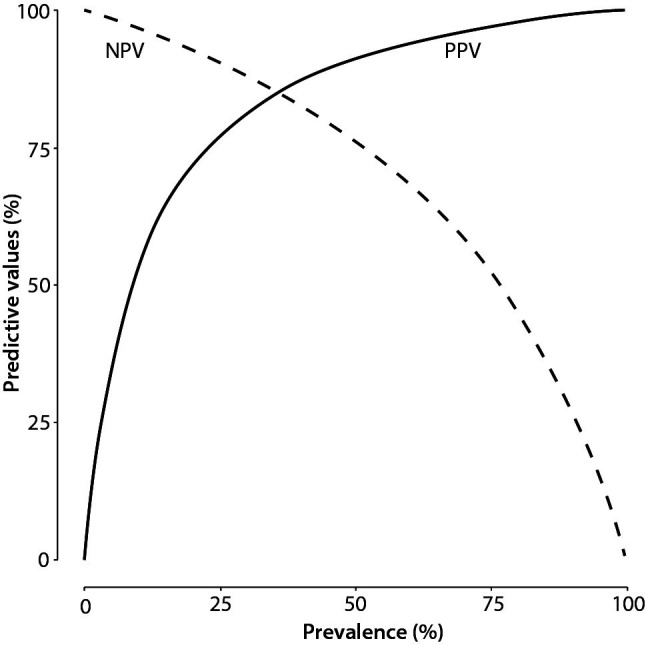
Variation of the positive predictive value (solid line) and negative predictive value (dashed line) with changes in disease prevalence in a test with a sensitivity of 0.72 and specificity of 0.93.

The Se and Sp are probably the most common performance indices used to assess a diagnostic test. They are characteristics of the test and are theoretically not supposed to be changed for a test with dichotomous result, or for a given cut-off value for tests with continuous results (*5*). While the test Se and Sp are very useful for laboratory specialists, they are not that useful for clinicians. In fact, most of clinicians do not understand them and interpret them inappropriately for a clear reason. In most instances, clinicians do not want to know the probability of a positive test in a diseased-person, *P*(*T*^+^ | *D*^+^), the *Se* (Eq. 2); instead, they are interested in knowing the probability that a person has a disease if his test is positive, *P*(*D*^+^ | *T*^+^) - the inverse probability of the *Se* (*4*). This inverse probability is termed the positive predictive value (PPV) and can be calculated as follows (Table 1):


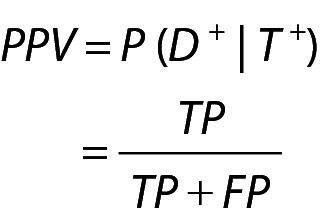
,

Similarly, we can define the negative predictive value (NPV), the probability that a person does not have a certain disease when the test is negative, P(D^–^ | T^–^)  -  the inverse probability of the Sp (*4*) - as follows (Table 1):


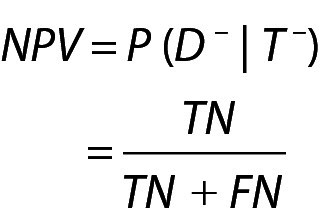
,

It can be shown that the PPV and NPV depend not only on the test Se and Sp, but also on the prior (also termed pre-test) probability (*pr*) of the disease of interest (the probability of the disease before we have any information about the test results; in the absence of any information, it is the prevalence of the disease), according to the following equations:


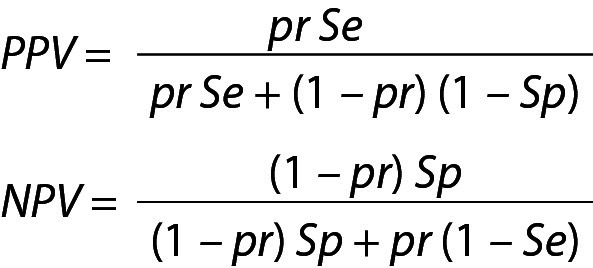
,

which implies that unlike the Se and Sp, which are constant for a certain test, PPV and NPV would vary from place to place depending on the *pr*. For a given test (with constant Se and Sp), PPV increases with increasing *pr*, while NPV decreases (Figure 1). This has profound consequences. Let us examine the situation through a case study.

## Case study

Suppose we want to determine whether an 80-year-old man who presented to our clinic with dysuria is likely to have prostate cancer or not. We requested to measure the serum prostate specific antigen (PSA). Assume that the result was 5.1 µg/L (for simplicity, assume that the test cut-off is 4.0 µg/L). The result is therefore interpreted as “positive.” Given a cutoff value of 4.0 µg/L, the PSA test has a Se of 0.72 (or 72%) and Sp of 0.93 (or 93%) (*6*). If we assume that the *pr* of the cancer in 80-year-old men hovers around 80%, then the PPV and NPV are 97% and 53%, respectively (Figure 1). Now suppose the same result was obtained for a 25-year-old man. The disease is very rare in 25-year-old men; assume that the *pr* is only 0.5%. All these translate into a PPV and NPV of less than 5% and more than 99%, respectively (Figure 1). Note that in both of these situations the Se and Sp of the PSA test remained unchanged.

Over years, practicing physicians learn about the probabilities of the disease conditions in their own settings and intuitively use the information to interpret the test results they ordered. That is why a general practitioner and a cardiologist would interpret the results of a single cardiac test, say, serum troponin I, differently, merely because they have different values for the prior probability, *pr*, of the myocardial infarction in their clinics.

## The probability, odds and the likelihood ratios

Another way to state the likelihood of an event (*e.g.*, to have a disease or not) is by calculating its odds, very prevailing in gambling. By definition, the odds of an event are the probability of that event happening divided by the probability of that event not happening (*7*). Therefore, the odds of having a disease can be calculated as follows:


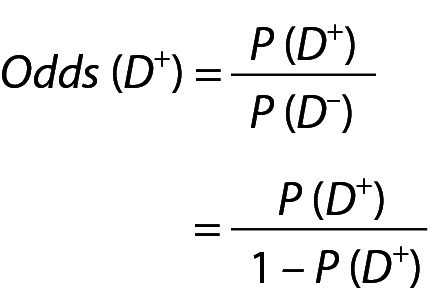
,

The probability of a certain disease before having any knowledge about any test results (technically, the disease *pr*, in the absence of any information) is termed, the prior or pre-test probability. After knowing the test result, we would revise the probability to arrive at a new value, the so-called *posterior* (also termed *post-test*) probability. We can define the posterior (post-test) and prior (pre-test) odds, accordingly. It can be shown that these odds are related according to the following equation, the Bayes’ formula (*8*, *9*):


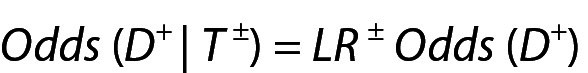
,

The left side of the above equation is the posterior odds of the disease given a positive or negative test result; the right side is the product of the prior odds of the disease and a constant, the positive likelihood ratio (LR^+^) or the negative likelihood ratio (LR^–^) (*4*, *9*). The LR^+^ and LR^–^ are calculated as follows:


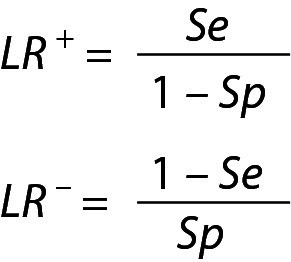
,

Therefore, given the Se and Sp for our case study, we have an LR^+^ of 10.3 and LR^–^ of 0.3 (Eq. 9). Let us try to interpret the test results in light of the likelihood ratios, we calculated.

First of all, we need to compute the odds corresponding to each of the prior probabilities of prostate cancer in each age  - 80% in the 80-year-old and 5% in the 25-year-old men, which are 4.0 (= 0.8 / 0.2) and 0.05 (≈ 0.05 / 0.95, note that for small values the odds are almost equal to the corresponding probability). Because the PSA test was found positive (5.1 µg/L), we then use the LR^+^ (had the test been negative, we would have used the LR^–^, instead). The posterior odds for the 80-year-old man is (*9*):


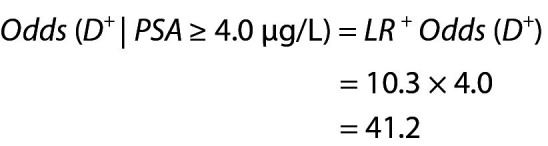
,

Therefore, given a positive PSA test, the odds of prostate cancer of 4.0 increased to 41.2, corresponding to a probability of 0.98 (98%). To calculate the probability from the odds, use the following equation, which can easily be derived from Eq. 7:


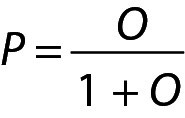
,

where *P* represents the probability; and *O*, the odds. For the 25-year-old man, the odds increased from 0.05 to 0.52, corresponding to a posterior probability of 0.34 (34%). Had the test been negative, say 3.1 µg/L, we would have used the *LR*^–^ of 0.3. Then, the odds of the prostate cancer for the 80-year-old man decrease from the prior odds of 4.0 to the posterior odds of 1.2 (= 0.3 x 4.0), corresponding to a posterior probability of 55% (Eq. 11).

Likelihood ratios, also fixed values for a given test, are also two test performance indices that are less commonly used by practitioners in their daily practice. Most of practicing physicians do not like working with probabilities and odds.

## Number needed to misdiagnose

So far, to assess the performance of a given test, we examined at least two indices concomitantly (Se and Sp, PPV and NPV, or LR^+^ and LR^–^). The number needed to misdiagnose (NNM) of a diagnostic test is defined as the number of people who need to be tested in order to find one misdiagnosed (with either a FP or a FN result); the index is calculated as follows (*10*):


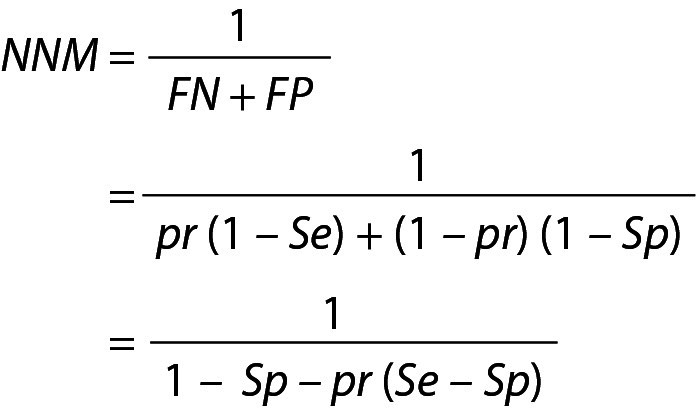
,

Like predictive values, the NNM depends on *pr*, and thus would vary from place to place, depending on the prior probability of the disease of interest. Plugging in the values from our case study, the NNM for the 25-year-old man (Eq. 12), will then be:


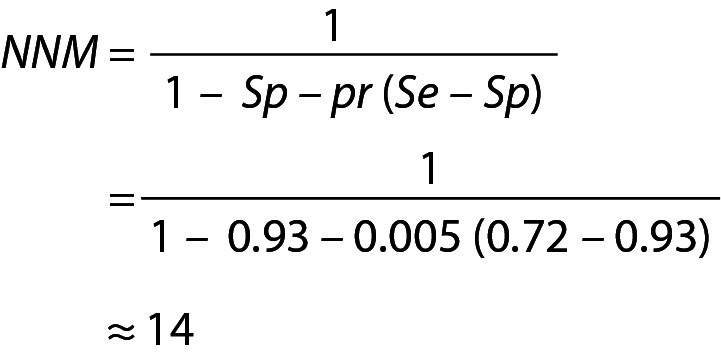
,

which means that one out of fourteen 25-year-old men tested is misdiagnosed (either FP or FN result). Obviously, tests that are more effective have higher NNM (*10*). I believe that NNM is superior to other indices hitherto discussed because it is readily comprehensible to clinicians. Furthermore, in contrast to the Se and Sp or PPV and NPV, which should be interpreted in conjunction with one another, the NNM is a single index, making it particularly straightforward to interpret. For example, a study on the diagnosis of biliary atresia revealed that the NNM for serum matrix metalloproteinase-7 was 25 (*i.e.*, 1 of 25 patients is misdiagnosed), while the index for gamma-glutamyltransferase was 3 (*i.e.*, 1 of 3 patients is misdiagnosed) (*11*). These findings show the superiority of the former test over the latter.

In the calculation of the NNM, it was assumed that a FN result has the same impact as a FP result may have. However, it is not the case in most instances. If we represent the cost of a FN relative to a FP result by C, then the weighted NNM (wNNM) can be defined as follows (*5*):


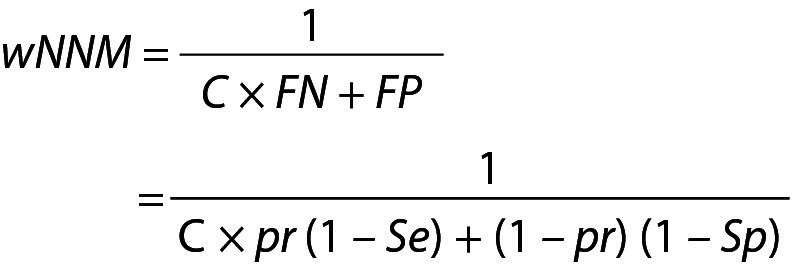
,

Unlike the NNM, which is readily comprehensible and clinically tangible, the wNNN is not as straightforward to understand. Nevertheless, it can readily be employed as a cost function in a variety of optimization problems, such as identifying the optimal cut-off value for a test with continuous results (*5*).

The number needed to diagnose (NND) is another test performance index. It is defined as the number of patients to be examined in order to correctly identify one person with the disease of interest (*12*). It can be calculated as follows:


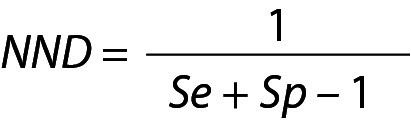
,

## Tests with continuous results

In our case study, we considered a PSA value equal or more than 4.0 µg/L “a positive test result.” This value is called the cut-off value. The cut-off value for a test with continuous results categorizes the results into either “positive” or “negative” results. Having a binary outcome, we can easily apply what we have so far discussed about the test performance indices to tests with continuous results (*5*). But, the choice of the cut-off value affects the test Se and Sp. Assuming that higher test values are more likely to be observed in those with the disease, an increase in the test cut-off value is associated with a decrease in the test Se and an increase in the test Sp (*5*). To achieve optimal performance, it is thus of paramount importance to set the cut-off value appropriately (*5*, *13*). One of the most commonly used methods to determine the most appropriate test cut-off value is the receiver operating characteristic (ROC) curve analysis (*5*).

There is a trade-off between the test Se and Sp. The ROC curve is a graphical representation of this trade-off; the graph plots the TP rate (*Se*) against the FP rate (1 – Sp) for each cut-off value (*5*). The ROC curve is confined to a unit square; the right-upper corner (Se = 1, Sp = 0), a point on the curve corresponds to the lowest possible cut-off value. With increasing the cut-off value, the test Se decreases and Sp increases, corresponding to moving on the curve from the right-upper corner down and to the left to the left-lower corner of the square where Se = 0 and Sp = 1, the point on the curve corresponding to the highest possible test cut-off value (*5*). The slope of the line connecting the left-lower corner of the ROC curve to the point corresponding to a certain cut-off value represents the LR^+^ (Eq. 9) associated with that cut-off value (*9*).

The area under the ROC curve (AUC) is another diagnostic test performance index; the AUC is the probability that the test result measured in a randomly selected person with the disease is higher than that measured in a disease-free individual (*7*). The AUC varies from 0.5 (for an uninformative test; *e.g.*, tossing a fair coin for the diagnosis) to 1.0 (for the gold standard test) (*5*, *14*).

One important point, which is sometimes not acknowledged by clinicians, is that the cut-off value is generally not the upper limit of the reference range of a test (*15*, *16*). It is different; the cut-off may be lower or higher than the upper limit of the reference range. For instance, a study conducted in Taiwan revealed that the upper limit of the reference range of PSA for men aged 60-69 years is 5.6 µg/L, while a PSA concentration of 4.0 µg/L is generally considered the cut-off value (*17*). To make a decision based on a test result, we need to consider the test cut-off value, not the reference range (*15*).

## Using diagnostic tests in research

Like other research studies, diagnostic accuracy studies are also at risk of bias. The main sources of bias in diagnostic accuracy studies originate in methodological flaws - inappropriate participant recruitment, wrong execution of the test, and mistakes in the interpretation of results (*18*). Although based on Eq. 1 and 3, the values of the test Se and Sp should theoretically be considered independent of the disease prevalence, they are not. These indices are typically obtained from diagnostic accuracy studies; and the choice of the gold standard test used, the choice of patients and disease-free people studied, among other factors, would affect the calculated Se and Sp (*19*). Therefore, the Se and Sp of a certain test in a clinical setting would be somewhat different from that reported in a study. An umbrella review of 23 meta-analyses (a total of 416 studies) revealed that for a certain diagnostic test, an increase in the prevalence of the disease of concern is associated with a lower test Sp and almost no change in Se (*20*).

Many research studies, including all seroprevalence studies, rely on the results of diagnostic tests. Since not all tests utilized are perfect (the gold standard test) and the test results may be FP or FN in certain cases, researchers should correct their findings to figure out unbiased estimates. For example, the seroprevalence of SARS-CoV-2 cannot reflect the true prevalence of COVID-19, given that the serology tests commonly used in such studies may provide FP or FN results (*21*). Is it necessary to always use gold standard tests in our research studies? Fortunately, no; there are simple solutions to solve this issue; researchers should be aware of the problem and the solution (*22*). For example, in a seroprevalence study aiming at detecting *Brucella canis* infection among dogs in Egypt, a combination of 2-mercaptoethanol (2-ME) tube agglutination test and rapid slide agglutination test was used (*23*). The study revealed an apparent seroprevalence of 3.8%. However, given that the combined Se and Sp of the battery of tests used are 28.1% and 99.9%, respectively, the authors correctly reported the true prevalence of the infection (13.2%) after plugging the values in the following equation (*22*, *24*):


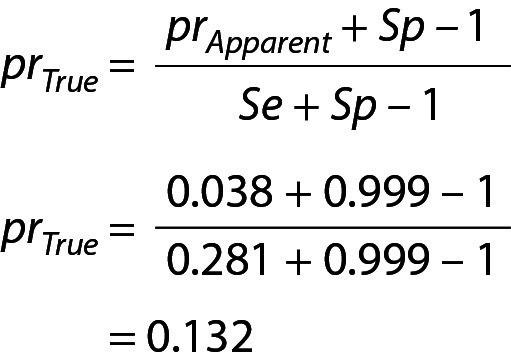
,

Another type of research studies are those conducted to determine the Se and Sp of a new test against the gold standard test. As mentioned earlier, the gold standard test may not always be readily available. Should a new test be always tested against a gold standard test? Here again, the answer is fortunately “no”.

To determine the Se and Sp of a new test, it is not necessary to always compare its results against those of a gold standard test. The results may be compared against a standard (not necessarily a gold standard) test with known Se and Sp, based on which it is possible to calculate the Se and Sp of the new test (*2*). Suppose that we compared the results of a test (T_2_) against another standard test (T_1_) with known Se of 85% and Sp of 90% (not a gold standard test, of course). Furthermore, assume that 25% of the studied people had a positive T_2_, the apparent prevalence of the condition of interest; and that the Se and Sp of T_2_ against T_1_ were 71% and 77%, respectively. Using the following equation, it is possible to calculate the Se and Sp of T_2_, had its results been compared against the gold standard test (*2*):


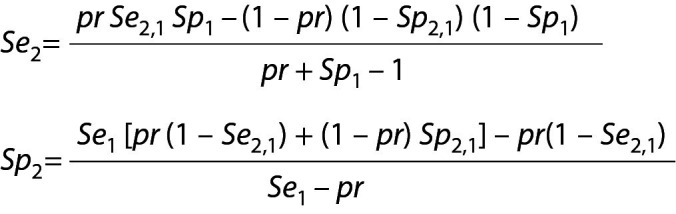
,

where *pr* is the apparent prevalence; Se_1_ and Sp_1_, the Se and Sp of T_1_ against the gold standard test; Se_2,1_ and Sp_2,1_, the Se and Sp of T_2_ against T_1_; and Se_2_ and Sp_2_, the Se and Sp of T_2_ had the test results been compared against the gold standard test. Plugging in the values, yield:


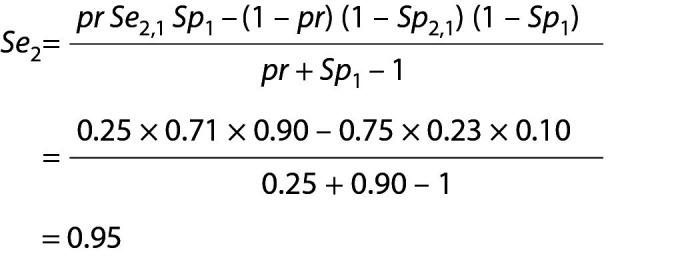
,


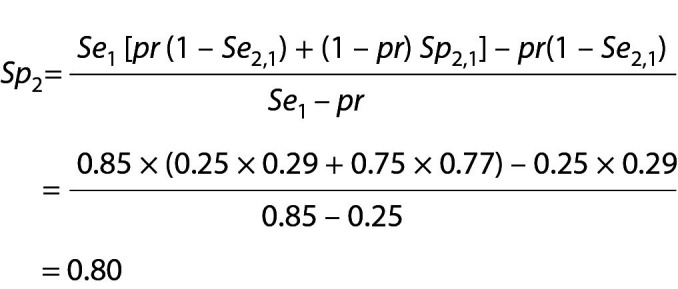
,

The true Se of T_2_ is 95%, while its true Sp is 80%.

Finally, it seems that it is possible to harvest the test performance indices merely based on the distribution of the test results in a large group of people (*21*, *25*, *26*). In fact, there are more indices hidden in the distribution. This is of particular application when there is no well-defined definition for a condition (*e.g.*, hypertension or diabetes). In this approach, using a mixture model, two hidden classes of people with and without the condition of interest are assumed. Using a non-linear regression, the parameters of the model will be computed. Based on these parameters, the test performance indices, the most appropriate cut-off value, and the prevalence of the condition of interest can readily be calculated (*21*, *25*, *26*). Details of the method are beyond the scope of this review.

## Conclusion

Of the common test performance indices, the test Se and Sp are not very useful for clinicians; PPV and NPV are much more comprehensible and useful for clinicians. The disadvantage of using PPV and NPV is that they should be interpreted together. The number needed to misdiagnose, a relatively new index, is both readily understandable by clinicians and easy to interpret, and may thus be considered a better index. To arrive at a correct estimation of test performance indices, it is of utmost importance to use a properly designed accuracy study and to take into account various aspects that could potentially compromise the validity of the study, including the choice of the gold standard and the population study, among other things.

## Data Availability

All data are presented in the article.
